# Supplementary material for: Patterns of Intron Gain and Loss in Fungi
Source: PLoS Biol. 2004 Nov 30;2(12):e422. doi: 10.1371/journal.pbio.0020422 (PMC532390; doi:10.1371/journal.pbio.0020422)
Supplement: Table S1 — Also available at http://genes.mit.edu/NielsenEtAl/. (4.3 MB ZIP). [file pbio.0020422.st001.zip › NielsenEtAl/html/1416.html]

AN3940.1.NCU09261.1.MG01611.1.FG00482.1


```
 CLUSTAL W (1.82) Multiple Sequence Alignments - Introns Inserted


Sequence 1: MG01611.1	221 aa
Sequence 2: FG00482.1	270 aa
Sequence 3: NCU09261.1	221 aa
Sequence 4: AN3940.1	270 aa
Alignment Length: 320 aa
Number Identitical Residues: 82 aa
Alignment Score (without introns) 4584


MG01611.1 	-MEIDDILREVDPDFDALP-SERRDLQLLTRAWVAERSAPELLE2WPSDGLFERVNDRIK
NCU09261.1	-MDIDDILREVDPVSHQIP-QETRDLQDLTRAWVAERSTPELLP~WPSNGLFERINDKIK
FG00482.1 	MMDIDDILRQVDPSSHGVP-SEARDLQALTRLWVAERSAPELLE2WPTDGLFERVNARIK
AN3940.1  	-MEIDDILASVDHTDISSPESTALDHQLLTRFWVAERSVPELLP~WPAR-LMERMMERVR
          	 *:***** .**      *..   * * *** ******.****  **:  *:**:  :::

MG01611.1 	QQ~IEHVEDMTGN--------MDPKTNFALIVIQTELERYKFLVRSYLRARMAK0IDKHT
NCU09261.1	RQ~IEKVEEMTGD--------MDPKTNFALIVIQTELERFKYLVRSFLRTRIAK0IDKHL
FG00482.1 	TQ~IEKVEDMTGD--------MDPKTNFALIVIQTELERYKFLMRSFLRARLAK0IDKHT
AN3940.1  	QQ0IETIEDLAAASSEPTSSTVNPNATLTLSILQTDLSRSQYLLRSLLRNRLSK~LTKHS
          	 * ** :*:::. ::..::::::*::.::* ::**:*.* ::*:** ** *::* : ** 

MG01611.1 	LHYLSTPSLR---------------------------------SRLSGTELAYATRHQAL
NCU09261.1	LHYISNDNLR---------------------------------TRMSQIEEAYARRHQLL
FG00482.1 	LHYLSSQELR---------------------------------DRLSPTELSYATKHQAL
AN3940.1  	MHYLLLSSHNPPNPPPASGSSEAKYQTPEDSVPLPDPDNLPRSSPLSPSETTFLYTHQQL
          	:**:   . .......::.::.:. .:...: . ..... . :  :*  * ::   ** *

MG01611.1 	LHNHYLSSFLASFPPALQNLNDTAGNISMIDTPDLDSAVFIRLLRDAFVE------GKGT
NCU09261.1	LHNHYLNSFLGTFPPAMQNLNDTAGNISMIDSPDLDSAVFIRLLRDAEIE------CHGV
FG00482.1 	LHNHYLSSFLASFPQQLQNLNDTAGNISMIDSPDLDTAVFIRMLRDKDVY------GKGT
AN3940.1  	LAKHFGSSFLNSFPPQLRRLDDNAGGTSMVQGPDTREAVFVRCLADEVLLLAPPGPGEGD
          	* :*: .*** :**  ::.*:*.**. **:: **   ***:* * *  :  :.... .* 

MG01611.1 	DSDGAMEGRD--GDILILR~---------~------------------WSDAKHLVD---
NCU09261.1	DVDGVLTGKE--GDIVILR~---------~------------------WSDAKGLVE---
FG00482.1 	DADITLPATN--GDVLIIR2VIVEMLSSR2HSKSTLSMLTIYCVVTCDKDDPNRKINTTC
AN3940.1  	DGEEVYGGTMRMGDVWVVR~---------~------------------WEAVKEAWE---
          	* : .  .    **: ::*                              .  :   :   

MG01611.1 	---------EGNAELV-------~--
NCU09261.1	---------KGDAELV-------~--
FG00482.1 	HGYNSKIALNGYAAMRRDDGETV1KA
AN3940.1  	---------RGDVEVL-------~--
          	         .* . :
```
